# Asymmetric DNA methylation of CpG dyads is a feature of secondary DMRs associated with the *Dlk1*/*Gtl2* imprinting cluster in mouse

**DOI:** 10.1186/s13072-017-0138-0

**Published:** 2017-06-21

**Authors:** Megan Guntrum, Ekaterina Vlasova, Tamara L. Davis

**Affiliations:** Department of Biology, Bryn Mawr College, 101 N. Merion Avenue, Bryn Mawr, PA 19010-2899 USA

**Keywords:** Genomic imprinting, DNA methylation, 5-Hydroxymethylcytosine, *Gtl2*, Gametic DMR, Somatic DMR, Epigenetics

## Abstract

**Background:**

Differential DNA methylation plays a critical role in the regulation of imprinted genes. The differentially methylated state of the imprinting control region is inherited via the gametes at fertilization, and is stably maintained in somatic cells throughout development, influencing the expression of genes across the imprinting cluster. In contrast, DNA methylation patterns are more labile at secondary differentially methylated regions which are established at imprinted loci during post-implantation development. To investigate the nature of these more variably methylated secondary differentially methylated regions, we adopted a hairpin linker bisulfite mutagenesis approach to examine CpG dyad methylation at differentially methylated regions associated with the murine *Dlk1/Gtl2* imprinting cluster on both complementary strands.

**Results:**

We observed homomethylation at greater than 90% of the methylated CpG dyads at the IG-DMR, which serves as the imprinting control element. In contrast, homomethylation was only observed at 67–78% of the methylated CpG dyads at the secondary differentially methylated regions; the remaining 22–33% of methylated CpG dyads exhibited hemimethylation.

**Conclusions:**

We propose that this high degree of hemimethylation could explain the variability in DNA methylation patterns at secondary differentially methylated regions associated with imprinted loci. We further suggest that the presence of 5-hydroxymethylation at secondary differentially methylated regions may result in hemimethylation and methylation variability as a result of passive and/or active demethylation mechanisms.

**Electronic supplementary material:**

The online version of this article (doi:10.1186/s13072-017-0138-0) contains supplementary material, which is available to authorized users.

## Background

Genomic imprinting in mammals results in the parent of origin-specific monoallelic expression of a subset of genes. Achieving the appropriate balance of gene expression from the maternally and paternally contributed genomes via the establishment of parental allele-specific imprinting marks is crucial for normal growth and development. Therefore, it is critical to understand the mechanisms responsible for controlling the expression of imprinted genes. To date, approximately 150 mammalian genes have been identified as imprinted [1, 2]. Most imprinted genes are found within clusters that contain a CpG-rich imprinting control region (ICR) that functions both to specify parental origin and to regulate imprinted expression of the genes within the cluster [3, 4]. Monoallelic expression of imprinted genes is achieved via multiple mechanisms, including epigenetic modifications such DNA methylation and histone modifications, as well as the activity of long noncoding RNAs [3, 4].

Differential DNA methylation at imprinted loci has been shown to play an important role in distinguishing the parental alleles and regulating their expression [5–9]. Differentially methylated regions (DMRs) associated with imprinted genes fall into two categories: primary and secondary DMRs. Primary, or gametic, DMRs serve as imprinting control regions (ICRs), functioning both to specify parental origin and as a shared regulatory element that controls the expression of genes throughout the associated imprinting cluster. Primary DMRs acquire DNA methylation on one of the two parental alleles during gametogenesis and remain differentially methylated from fertilization throughout development, thereby marking parental origin [3]. The differentially methylated state of primary DMRs can affect expression in a variety of ways. For example, primary DMRs can regulate gene expression through their differential ability to bind enhancer blocking proteins, thereby influencing the activity of an insulator [10, 11]. In other cases, primary DMRs are located at promoters, where they have been shown to directly influence the expression of long noncoding RNAs that subsequently regulate the expression of other genes in the imprinting cluster [12–15]. In contrast, secondary DMRs acquire parent of origin-specific DNA methylation after implantation [16–19]. Secondary DMRs are generally located at promoters or within gene bodies, and the acquisition of parental allele-specific DNA methylation at these sequences is dependent on differential methylation of the associated ICR, while the converse is not true [8, 9, 20]. While secondary DMRs do not function as primary imprinting marks, methylation of these regions frequently corresponds with gene silencing and may play a role in maintaining imprinted expression [17, 21–23].

The DNA methylation associated with primary DMRs is very stable, with the methylated allele displaying 90–100% methylation at the cytosines located in CpG dinucleotides throughout development [5, 19, 24–27]. DNA methylation at secondary DMRs is less consistent. For example, methylation at secondary DMRs located at the *H19* and *Gtl2* promoters average 70 and 78.9%, respectively, as compared to methylation at their respective primary DMRs, which average ~90 and 95.8% [5, 28]. We recently illustrated that the highly variable DNA methylation pattern at the secondary DMR associated with the imprinted *Dlk1* gene is asymmetric, with 35% of the methylated CpG dyads displaying hemimethylation [18]. The trend that DNA methylation is more stable at primary DMRs than at secondary DMRs associated with imprinted genes has also been observed at human imprinted loci [29].

Our current study investigates the nature of secondary DMRs associated with imprinted loci and potential causes of methylation instability, such as a failure to maintain DNA methylation and/or active demethylation catalyzed by the TET enzymes [30–34]. To test the hypothesis that variably methylated secondary DMRs display higher levels of hemimethylation than stably methylated primary DMRs, we analyzed DNA methylation at two additional DMRs associated with the *Dlk1*/*Gtl2* imprinting cluster: the IG-DMR, a primary DMR, and the *Gtl2*-DMR, a secondary DMR [14, 28]. We also quantified the level of 5-hydroxymethylation (5-hmC) at the IG-, *Gtl2*- and *Dlk1*-DMRs to determine whether there is a correlation between high levels of hemimethylation and high levels of 5-hydroxymethylation. Our results support the hypothesis that high levels of 5-hmC may contribute to methylation instability at secondary DMRs associated with imprinted genes.

## Results

### CpG dyads within the *Gtl2*-DMR display high levels of hemimethylation

**Fig. 1:**
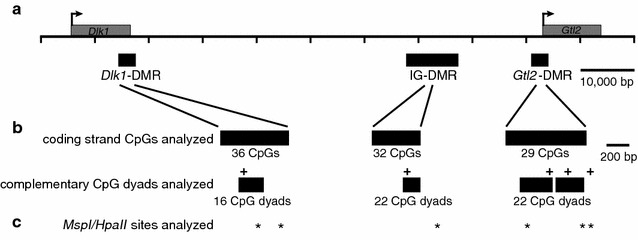
Schematic representing sequences analyzed within the *Dlk1*/*Gtl2* imprinting cluster. **a** *Dlk1/Gtl2* imprinting cluster on mouse chromosome 12, including transcriptional start sites (*arrows*), transcription units (*gray boxes*) and differentially methylated regions (*black boxes*). **b** Regions of the *Dlk1*-, IG- and *Gtl2*-DMRs analyzed by bisulfite mutagenesis and DNA sequencing. Information regarding coding strand CpGs, which were analyzed in previous studies [18, 28] provides context for the current analyses. The regions in which complementary CpG dyads were analyzed at the *Dlk1*-, IG- and *Gtl2*-DMRs are 156 bp, 126 bp and 520 bp, respectively, and correspond to positions 109,459,709-109,459,865, 109,528,345-109,528,471 and 109,541,256-109,541,776 (GenBank Accession Number NC_000078.6). Sequence polymorphisms used to distinguish the parental alleles (+); genomic coordinates are listed in the Methods. **c** *Msp*I/*Hpa*II sites analyzed for 5-methylcytosine and 5-hydroxymethylcytosine (*); genomic coordinates are listed in “Methods”

To determine whether asymmetric methylation is unique to the *Dlk1*-DMR or is a feature of other secondary DMRs associated with imprinted loci, we examined CpG dyad methylation at the linked *Gtl2*-DMR. We had previously analyzed DNA methylation on the coding strand of the *Gtl2*-DMR and observed moderate variability in the methylation status, with the 5′ half of the region analyzed displaying lower average DNA methylation levels than the 3′ half [28]. We therefore assessed the DNA methylation status of cytosines located in 22 pairs of complementary CpG dinucleotides spanning this region to determine whether these CpG dyads were homomethylated versus hemimethylated (Fig. 1). All of our experiments were conducted using F_1_ hybrid tissues collected from crosses between C57BL/6 (B6) and a specially derived strain containing *Mus musculus castaneus* (CAST)-derived sequences from chromosome 12 on an otherwise C57BL/6 genetic background (CAST12) [18, 28], allowing us to distinguish paternally inherited alleles from maternally inherited alleles based on sequence polymorphisms (detailed in the “Methods”).

**Fig. 2:**
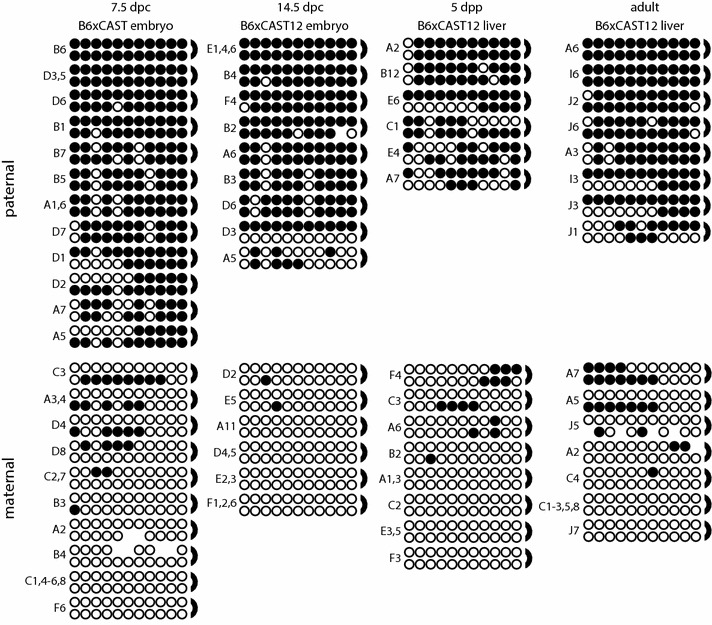
DNA methylation in the 5′ portion of the *Gtl2*-DMR displays a high level of hemimethylation. Bisulfite mutagenesis and sequencing of F_1_ hybrid DNA derived from 7.5 d.p.c. B6 × CAST embryos, 14.5 d.p.c. B6 × CAST12 embryos, 5 d.p.p. B6 × CAST12 liver and adult B6 × CAST12 liver. Individual *circles* in each *row* represent one of the 11 potentially methylated CpG dinucleotides analyzed, and each paired *row* of *circles* represents the complementary strands of an individual subclone; *semicircles* to the *right* indicate the location of the linker connecting the complementary strands. *Filled circles* represent methylated cytosines, *open circles* represent unmethylated cytosines, and absent *circles* represent ambiguous data. Labels to the *left* identify the PCR subclone analyzed; *letters* represent independent amplification reactions, while *numbers* represent individual subclones. Subclones derived from the same amplification that have identical sequence and methylation patterns are grouped together, as it was not possible to determine whether these amplicons were derived from the same or different template molecules

**Fig. 3:**
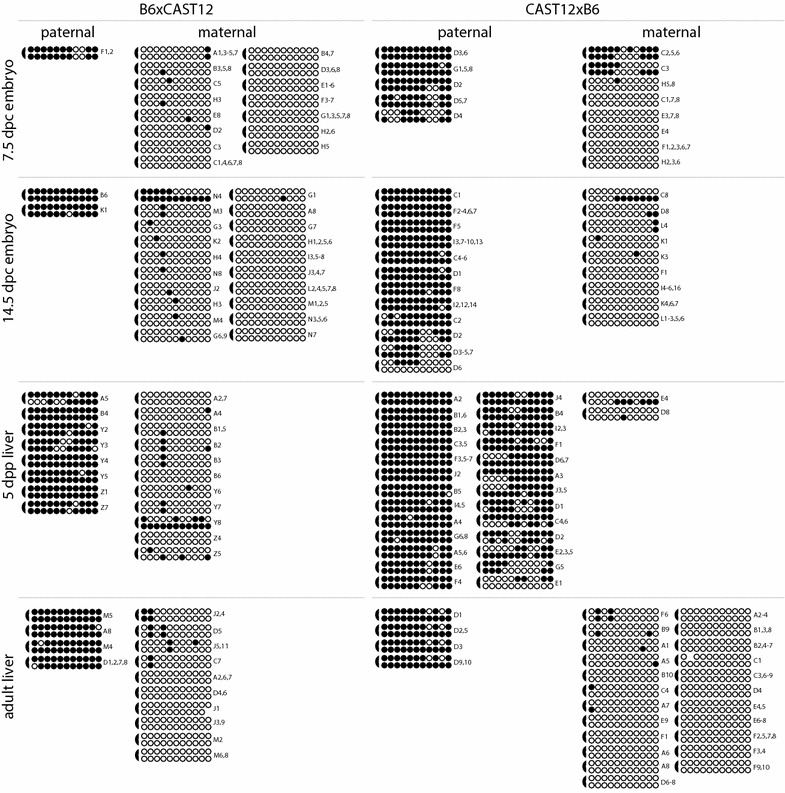
DNA methylation in the 3′ portion of the *Gtl2*-DMR displays a high level of hemimethylation. Details as described in Fig. 2, with the following exceptions: *semicircles* indicating the location of the linker connecting the complementary strands are on the *left* and labels identifying PCR subclones analyzed are on the *right*. The bias in amplification of paternal versus maternal strands was inconsistent and was not dependent on developmental stage nor F_1_ hybrid genetic background

We analyzed CpG dyad methylation in DNA derived from four developmental stages: 7.5 d.p.c. embryo, 14.5 d.p.c. embryo, 5 d.p.p. liver and adult liver. The DNA methylation patterns on each parental allele were consistent throughout development and were also similar in tissues obtained from reciprocal crosses (Figs. 2, 3). Across all four developmental stages, cytosines in CpG dinucleotides were methylated 80-93% of the time on paternal alleles and 6–10% of the time on maternal alleles (Table 1). We assessed the significance of these results at each developmental stage using a Mann–Whitney *U* test and found that the median level of DNA methylation was significantly higher on the paternal alleles as compared to the maternal alleles in all of the tissues examined, with *P* values ranging from <0.0001 to 0.0147 (Table 2; Additional File 1). Furthermore, *P* values derived from Mann–Whitney *U* tests illustrate that median DNA methylation levels did not vary significantly across development on either the paternal or the maternal allele (Additional File 1). Average DNA methylation levels did not vary substantially between the 5′ half versus the 3′ half of the analyzed region. These results confirm that the *Gtl2*-DMR is differentially methylated throughout development.

**Table 1:** Average levels of DNA methylation on paternal and maternal alleles at the *Dlk1*-, IG- and *Gtl2*-DMRs across four developmental stages

		*Dlk1*-DMR		IG-DMR		*Gtl2*-DMR, 5′	*Gtl2*-DMR, 3′	
		BxC	CxB	BxC	CxB	BxC	BxC	CxB
% methylation (# methylated/total)	P	42.5% (510/1199)	42% (541/1287)	96.1% (2960/3080)	95.5% (2982/3123)	79.5% (699/879)	92.7% (306/330)	85.9% (878/1034)
	M	16.6% (251/1516)	28.4% (367/1292)	11.2% (110/918)	12.2% (75/616)	9.5% (64/671)	6.2% (76/1231)	6.4% (59/923)
	total	28% (761/2715)	35.2% (908/2579)	76.8% (3070/3998)	81.8% (3057/3739)	49.2% (763/1550)	24.5% (382/1561)	47.9% (937/1957)
% homomethylation (# homomethylated/total)	P	79.5% (221/278)	70.6% (223/316)	94.2% (1436/1524)	92.9% (1436/1545)	76.3% (302/396)	88.9% (144/162)	82.5% (397/481)
	M	63.4% (97/153)	65.7% (140/213)	39.7% (31/78)	70.5% (31/44)	12.2% (7/57)	35.7% (20/56)	31.1% (14/45)
	total	73.8% (318/431)	68.6% (363/529)	91.6% (1467/1602)	92.3% (1467/1589)	68.2% (309/453)	75.2% (164/218)	78.1% (411/526)
% hemimethylation (# hemimethylated/total)	P	20.5% (57/278)	29.4% (93/316)	5.8% (88/1524)	7.1% (109/1545)	23.7% (94/396)	11.1% (18/162)	17.5% (84/481)
	M	36.6% (56/153)	34.3% (73/213)	60.3% (47/78)	29.5% (13/44)	87.7% (60/57)	64.3% (36/56)	68.9% (31/45)
	total	26.2% (113/431)	31.4% (166/529)	8.4% (135/1602)	7.7% (122/1589)	31.8% (144/453)	24.8% (54/218)	21.9% (115/526)

Percent methylation and number of sites analyzed on the paternal and maternal alleles in DNA derived from individual developmental stages (7.5 and 14.5 d.p.c. embryos and 5 d.p.p. and adult liver) are given in Additional file 2: Table S1. Data for the *Dlk1*-DMR were calculated from Gagne et al. [18]

**Table 2:** Average levels of DNA methylation on the paternal and maternal alleles are significantly different at the *Gtl2*- and IG-DMRs

	Genomic DNA sample	Grouped			Ungrouped		
		% methylation, paternal alleles	% methylation, maternal alleles	*P* value	% methylation, paternal alleles	% methylation, maternal alleles	*P* value
*Gtl2*-DMR, 5′, BxC	7.5 d.p.c. embryo	82.2% (217/264)	11.7% (25/214)	0.0001	83.4% (257/308)	9.2% (32/346)	<0.0001
	14.5 d.p.c. embryo	82.5% (235/285)	1.5% (2/132)	0.0024	84.8% (279/329)	0.9% (2/220)	0.0001
	5 d.p.p. liver	69.7% (92/132)	8% (14/176)	0.0024	69.7% (92/132)	6.4% (14/220)	0.0014
	adult liver	78.3% (155/198)	15.4% (23/149)	0.0021	78.3% (155/198)	9.7% (23/237)	0.0004
IG-DMR, BxC	7.5 d.p.c. embryo	94.2% (912/968)	1.5% (2/132)	<0.01	94.5% (1621/1716)	1.1% (2/176)	<0.01
	14.5 d.p.c. embryo	96.9% (597/616)	11.1% (39/352)	0.0002	97.6% (816/836)	9.8% (39/396)	<0.0001
	5 d.p.p. liver	96.8% (852/880)	17.4% (45/258)	0.0003	97.6% (1159/1188)	16.8% (58/346)	<0.0001
	adult liver	97.2% (599/616)	13.6% (24/176)	<0.01	97.7% (1333/1364)	13.6% (24/176)	<0.01

**Fig. 4:**
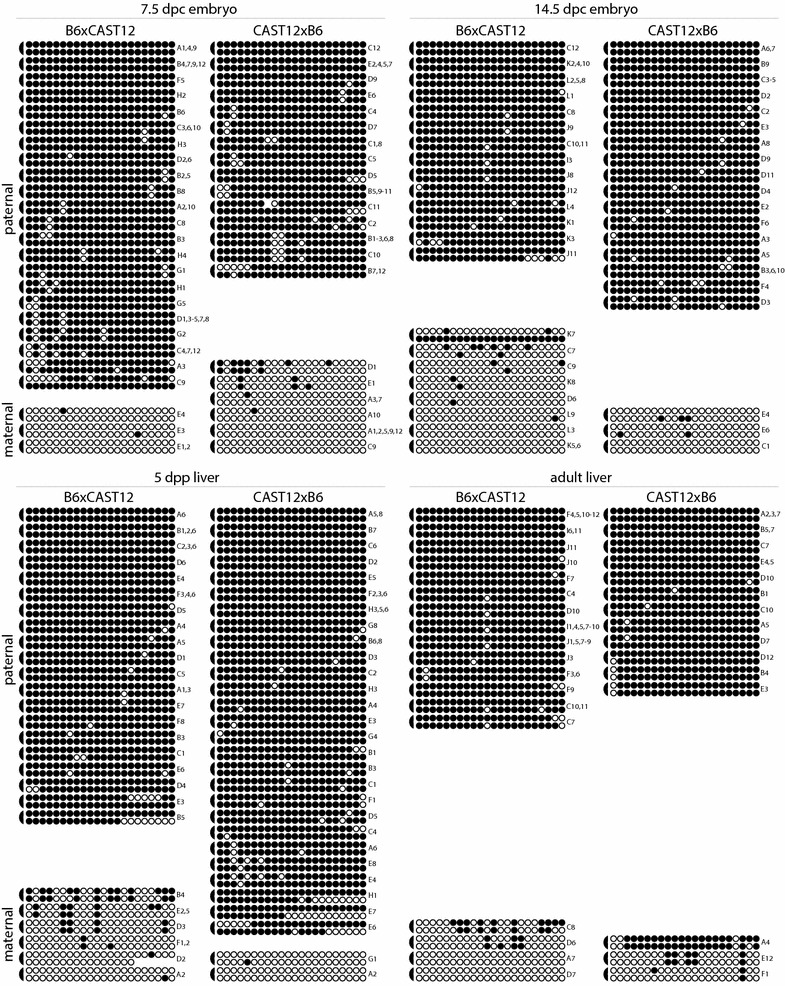
DNA methylation at the IG-DMR displays low levels of hemimethylation. Details as described in Fig. 2, with the following exceptions: *individual circles* in each row represent one of the 22 potentially methylated CpG dinucleotides analyzed, *semicircles* indicating the location of the linker connecting the complementary strands are on the *left* and labels identifying PCR subclones analyzed are on the *right*

Percent methylation and number of sites analyzed on the paternal and maternal alleles in DNA derived from 7.5 and 14.5 d.p.c. embryos and 5 d.p.p. and adult liver. Grouped data were derived when subclones from the same PCR with identical DNA methylation patterns and sequences were grouped as a single sample, as illustrated in Figs. 2, 4. Ungrouped data were derived when subclones from the same PCR with identical DNA methylation patterns and sequences were treated as independent samples. *P* values were calculated using a Mann–Whitney *U* test

Homomethylation was observed at 68–78% of the CpG dyads containing methylated cytosine, while hemimethylation was detected at 22–32% of these CpG dyads (Table 1). The levels of homo- and hemimethylation at the *Gtl2*-DMR were similar to the overall average of 65% homomethylation and 35% hemimethylation observed at the *Dlk1*-DMR [18]. When we restricted our analysis to the same four developmental stages assessed in this study, we observed 69–74% homomethylation at methylated CpG dyads within the *Dlk1*-DMR and 26–31% hemimethylation (Table 1). We tested the homo- and hemimethylation levels at the *Gtl2*- and *Dlk1*-DMRs for statistical independence by performing Chi-squared analysis and determined that hemimethylation levels are not significantly different at these loci (*P* = 0.1318). Therefore, we conclude that hemimethylation levels are similar at two distinct secondary DMRs located within the *Dlk1*/*Gtl2* imprinting cluster.

### CpG dyads within the IG-DMR display low levels of hemimethylation

We next assessed hemimethylation levels at the IG-DMR, which serves as the imprinting control region for the *Dlk1*/*Gtl2* imprinting cluster [19, 35]. We analyzed 22 CpG dyads located within the IG-DMR (Fig. 1). We had previously analyzed DNA methylation on the coding strand of this region and had found it to lack variability, with paternally inherited alleles showing near 100% DNA methylation and maternally inherited alleles displaying less than 10% DNA methylation [28]. Consistent with our previous findings, we observed methylation at 96 and 12% of paternally versus maternally inherited CpG dinucleotides located within the IG-DMR, respectively (Fig. 4; Table 1). The median levels of DNA methylation were significantly higher on paternally derived alleles as compared to maternally derived alleles for all tissues analyzed, with *P* values ranging from <0.0001 to <0.01 (Table 2; Additional File 1), confirming that this region is differentially methylated throughout development. There were no significant differences in the DNA methylation profile of maternal alleles across development (Additional File 1). In contrast, while median DNA methylation levels on paternal alleles was not significantly different between the 14.5 d.p.c. embryo, 5 d.p.p. liver or adult liver samples, the distribution of DNA methylation on paternal alleles derived from 7.5 d.p.c. embryos was different from the distribution in 14.5 d.p.c. embryos (*P* = 0.0021), 5 d.p.p. liver (*P* = 0.0178) and adult liver (*P* = 0.0006). The significance of these results may be attributed to the fact that paternal alleles derived from 7.5 d.p.c. embryos contain more unmethylated cytosines than paternal alleles derived from other developmental stages (Fig. 4; Additional File 1).

**Fig. 5:**
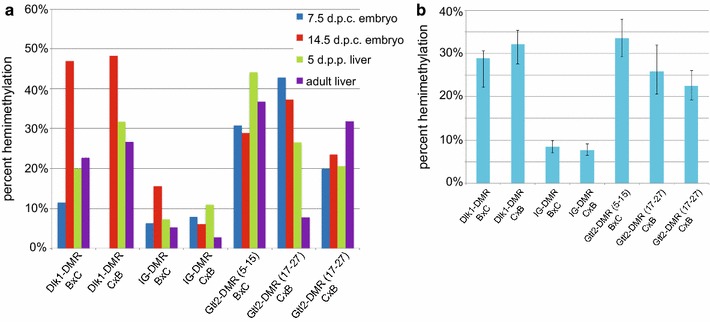
Hemimethylation levels are higher at the *Dlk1*- and *Gtl2*- secondary DMRs than at the primary IG-DMR. **a** Hemimethylation levels at the *Dlk1*-, IG- and *Gtl2*-DMRs in DNA from 7.5 and 14.5 d.p.c. embryos and from 5 d.p.p. and adult liver. **b** Across development, average hemimethylation levels at the IG-DMR are less than 8.5%, while hemimethylation averages range from 22 to 34% at the *Dlk1*- and *Gtl2*-DMRs. *Error bars* represent the 95% confidence interval

Of the CpG dyads displaying cytosine methylation within the IG-DMR, 92% were homomethylated, while 8% were hemimethylated (Table 1). The frequency of hemimethylation was higher on maternally inherited alleles, which were much more sparsely methylated than paternally inherited alleles (Table 1). On average, the level of hemimethylation we observed at the IG-DMR at each developmental stage and across development was lower than the level we observed at either the *Dlk1*-DMR or the *Gtl2*-DMR (Fig. 5). We assessed the significance of this result using a Chi-squared test of independence. The level of hemimethylation at the primary IG-DMR is significantly different than the level at either of the secondary DMRs (IG- vs. *Dlk1*-DMR, *P*-5.93 × 10^−65^; IG- vs. *Gtl2*-DMR, *P* = 8.76 × 10^−57^), supporting our hypothesis that high levels of hemimethylation are characteristic of secondary, but not primary, DMRs associated with imprinted loci.

For all of our DNA methylation analyses, we employed a conservative approach whereby we grouped subclones that were derived from the same PCR amplification and had identical sequence and DNA methylation patterns, as it is not possible to determine whether these amplicons are derived from the same or different template molecules. As it is possible that some of the grouped subclones actually represent independent samples, we performed the same analyses for ungrouped data sets in which each subclone was treated as an independent sample. Ungrouping the identical subclones resulted in greater significant differences between the parental alleles at both the *Gtl2*-DMR and the IG-DMR, confirming their differentially methylated status (Table 2; Additional File 1). While ungrouping identical subclones reduced hemimethylation values at both the *Gtl2*- and IG-DMRs, from 31.8 to 29.8 and 8.4 to 6.1%, respectively (Additional file 3: Table S2, Additional file 4: Table S3), the difference in hemimethylation levels between the primary IG-DMR and the secondary *Gtl2*-DMR remains highly significant as assessed using a Chi-squared test of independence (*P* = 2.81 × 10^−59^).

**Table 3:** Average 5-hydroxymethylation levels at *Msp*I sites located in the *Dlk1*-, IG- and G*tl2*-DMRs

	*Dlk1*, site A	*Dlk1*, site B	IG-DMR	*Gtl2*, site A	*Gtl2*, site B	*Gtl2*, site C
9.5 d.p.c. embryo (n)	21.5% (4)	6.6% (3)	2.9% (3)	0% (4)	1.4% (4)	0.79% (1)
14.5 d.p.c. embryo (n)	39% (3)	10% (3)	3.1% (3)	0.8% (3)	1.6% (3)	1.2% (1)
5 d.p.p. liver (n)	10.2% (8)	6.5% (8)	3.7% (3)	2.5% (4)	0.9% (6)	1.4% (1)
Adult liver (n)	8.1% (3)	1.7% (3)	8.7% (3)	1.9% (3)	0.4% (3)	2.4% (1)

n = number of biological replicates; each biological replicate was composed of three technical replicates; data for individual biological replicates are given in Additional file 5: Table S4

### High levels of hemimethylation correlate with high levels of 5-hydroxymethylcytosine at the *Dlk1*-DMR, but not at the *Gtl2*-DMR

We hypothesized that hemimethylation at the *Dlk1*- and *Gtl2*-DMRs could arise as a result of sequential oxidation of 5-methylcytosine (5-mC) by the TET enzymes followed by either passive depletion of methylation following DNA replication or thymine DNA glycosylase-mediated base excision repair, ultimately resulting in demethylation of that residue [30–34]. If the TET enzymes are responsible for demethylation of cytosines leading to high levels of hemimethylation at secondary DMRs associated with imprinted loci, we would expect to see 5-hydroxymethylcytosine (5-hmC), an oxidation intermediate in this pathway, at these loci [30, 31]. We therefore assessed the relative levels of 5-mC and 5-hmC at the *Dlk1*-, *Gtl2*- and IG-DMRs in genomic DNA isolated from 9.5 and 14.5 d.p.c. embryos and from 5 d.p.p. and adult liver. To conduct this analysis, we glucosylated genomic DNA derived from each of the four developmental stages listed above, digested glucosylated and unglucosylated samples with *Msp*I, *Hpa*II or no enzyme, amplified the resulting products using qPCR and calculated percent 5-hmC based on the method previously described by Magalhães et al. [36]. We used 9.5 d.p.c. embryos as our earliest developmental time point, rather than 7.5 d.p.c., in order to have sufficient DNA for these analyses.

At the IG-DMR, where we observed low levels of hemimethylation, we detected correspondingly low levels of 5-hmC in DNA derived from 9.5 to 14.5 d.p.c. embryos and from 5 d.p.p. liver: 3–4% of the methylation detected at the *Msp*I site located within the IG-DMR was 5-hmC (Table 3). Higher 5-hmC levels were detected in adult liver, which also had more variation between biological replicates (Additional file 5: Table S4). In contrast, our analysis of two *Msp*I sites located within the *Dlk1*-DMR detected 5-hmC levels ranging from 7 to 40% in embryos and neonatal liver, with highest levels observed in 14.5 d.p.c. embryos (Table 3). This higher level of 5-hmC at the *Dlk1*-DMR correlates with the high levels of hemimethylation detected at this locus. We therefore anticipated that we would observe similarly high levels of 5-hmC at the *Gtl2*-DMR, which also displays high hemimethylation levels. However, the level of 5-hmC at the *Gtl2*-DMR inversely correlated with hemimethylation levels at this locus. While we detected high levels of hemimethylation at the *Gtl2*-DMR, we observed very low levels of 5-hmC at three *Msp*I sites located within this locus (Table 3).

### 5-hmC is absent at the *Dlk1*-, *Gtl2*- and IG-DMRs in triple TET knockout ES cells

To validate the assay used to assess 5-hmC levels in the *Dlk1*-, *Gtl2*- and IG-DMRs, we conducted an analysis of 5-hmC in DNA derived from wild-type and triple TET knockout embryonic stem (ES) cells [37]. In wild-type ES cells, we detected 5-hmC at 12, 14 and 10% of the methylated cytosines located within *Msp*I sites at the *Dlk1*-, *Gtl2*- and IG-DMRs, respectively (Table 4). In ES cells with a triple knockout of the TET genes, 5-hmC was undetectable at all three loci (Table 4). These data validate that the assay employed to detect 5-hmC and confirm that the presence of 5-hmC at these loci is dependent on functional TET enzyme activity.

**Table 4:** 5-Hydroxymethylation at the *Dlk1*-, IG- and G*tl2*-DMRs in embryonic stem cells is dependent on the activity of TET enzymes

	WT ES cells (%)	TET-KO ES cells (%)
*Dlk1*-DMR, site A	12.0	0
IG-DMR	9.9	0
*Gtl2*-DMR	13.6	0

## Discussion

Differential DNA methylation plays an important role in the regulation of imprinted genes, directly affecting the activity of ICRs as well as directly or indirectly regulating the expression of genes within imprinting clusters [3 4 and references therein]. Stably maintaining DNA methylation at imprinted loci is critical for normal growth and development, and aberrant DNA methylation patterns are associated not only with abnormal expression of imprinted genes, but also with multiple imprinting disorders [38, 39]. Therefore, understanding the normal methylation patterns and how they are altered are important for elucidating the regulation of imprinted genes.

Investigation of DNA methylation at imprinted loci has identified a difference in methylation stability at primary versus secondary DMRs: methylation patterns at primary DMRs appear to be very stable and consistent, while methylation at secondary DMRs is more variable in both mice and humans [5, 18, 19, 24–29]. Our previous analyses identified a correlation between variable methylation patterns and high levels of hemimethylation at CpG dyads at the secondary DMR associated with the imprinted *Dlk1* gene [18]. To test the hypothesis that hemimethylation is a normal characteristic of secondary DMRs associated with imprinted loci, we utilized a hairpin bisulfite mutagenesis approach to examine CpG dyad methylation at another secondary DMR (the *Gtl2*-DMR) as well as at a primary DMR that serves as an ICR (the IG-DMR associated with the *Dlk1*/*Gtl2* imprinting cluster). We found high levels of hemimethylation at the *Gtl2*-DMR, but not at the IG-DMR, supporting our hypothesis (Figs. 2, 3, 4).

It is possible that some of the hemimethylation we observed resulted from errors associated with bisulfite mutagenesis, including failed conversion of unmethylated cytosines and inappropriate conversion of methylated cytosines [40]. Either one of these errors could lead to a hemimethylated CpG dyad. It is unlikely that these errors had an appreciable effect on the hemimethylation levels we observed, as the high molarity, high temperature, short reaction time methodology we employed have been shown to result in low bisulfite conversion error rates [40, 41]. To test this assumption, we directly calculated the failed conversion rate by examining 20,868 non-CpG cytosine locations in our bisulfite-treated samples. We identified 291 cytosines, some of which may have arisen as a result of PCR-induced error rather than failed conversion (Additional file 6: Table S5). The failed conversion rate we observed of 1.39% is similar to the error rate reported by Genereux et al. [40] for hairpin-linked molecules treated under high molarity, high temperature, 90-min reaction time mutagenesis conditions. We therefore conclude that the predicted and observed error rates are unlikely to account for an appreciable amount of the hemimethylation we observed, particularly at the secondary DMRs, where hemimethylation levels range from 22 to 35%. Furthermore, even if hemimethylation levels vary slightly due to conversion errors, it would not affect our overall conclusion that the difference in hemimethylation levels between primary and secondary DMRs is highly significant.

Based on the observation that secondary DMRs have high levels of hemimethylation, we further proposed that oxidation of 5-methylcytosine by the TET enzymes could result in hemimethylation either by impeding the activity of DNMT1, resulting in replication-dependent passive demethylation, or by further processing of the oxidized products 5-fC and 5-caC and their removal via base excision repair, a mechanism of active demethylation [30–34]. In support of this hypothesis, we found low levels of 5-hmC at the IG-DMR, which had correspondingly low levels of hemimethylation, and high levels of 5-hmC at the *Dlk1*-DMR, which had correspondingly high levels of hemimethylation. However, at the *Gtl2*-DMR, where we observed high levels of hemimethylation, we detected low levels of 5-hmC.

The fact that we observed low levels of 5-hmC at the *Gtl2*-DMR may be a consequence of our experimental approach. The earliest developmental stage at which we assessed 5-hmC was embryonic day 9.5, when methylation of the *Gtl2*-DMR is already relatively stable as compared to methylation at earlier embryonic stages such as 6.5 and 7.5 d.p.c. [28]. While we detected very little 5-hmC in DNA derived from 9.5 to 14.5 d.p.c. embryos and from neonatal and adult tissues, it is possible that the *Gtl2*-DMR contains high levels of 5-hmC earlier in development, when DNA methylation patterns at this locus are more labile. Alternatively, the absence of 5-hmC at the *Gtl2*-DMR may point to the relative importance of maintaining DNA methylation at this locus in order to appropriately silence *Gtl2* expression on the paternal allele and achieve appropriate imprinted expression patterns across the *Dlk1*/*Gtl2* imprinting cluster. Indeed, studies have shown that loss of methylation on the paternally inherited *Gtl2*-DMR correlates with expression of *Gtl2* from this allele [42, 43]. In contrast to the substantiated role of differential DNA methylation at the *Gtl2*-DMR, we and others have questioned whether differential methylation at the *Dlk1*-DMR, which is located in the 5th exon of *Dlk1*, plays an important regulatory role [18, 19].

To further investigate the hypothesis that 5-hmC contributes to DNA methylation instability at secondary DMRs associated with imprinted genes, we are currently examining CpG dyad methylation patterns and 5-hmC levels at additional imprinted loci. The work described herein focused on analyses of primary and secondary DMRs associated with the *Dlk1*/*Gtl2* imprinting cluster on mouse chromosome 12. Our current inquiries include an examination of CpG dyad methylation patterns at imprinted loci distributed across multiple genomic locations, including both paternally and maternally methylated DMRs, and our preliminary results suggest that the relationship between 5-hmC and DNA methylation variability at secondary DMRs may be generalizable (Davis Laboratory, unpublished data).

The presence of 5-hmC at secondary DMRs associated with imprinted genes suggests that the TET proteins play a role in modulating DNA methylation stability at these loci. Several studies have shown that the TET proteins play a role in epigenetic reprogramming throughout development [44 and references therein]. For example, the presence of 5-hmC at repressed promoters and in the gene bodies of expressed genes in ES cells is concomitant with the association of TET1 and TET2, respectively, with these sequences [45–48]. Recent reports have also illustrated the role of TET enzymes in epigenetic reprogramming during primordial germ cell development, including the erasure of imprinting marks [49–51], and that a double knockout of TET1 and TET2 disrupts normal methylation and expression patterns at imprinted loci [52]. Liu and colleagues further reported that some imprinted loci, such as *H19*, appear to be more sensitive to TET activity based on methylation patterns in wild-type *vs.* TET knockout (TET-KO) ES cells, suggesting that the TET proteins may have different effects at different imprinted loci [53]. Interestingly, the study conducted by Liu et al. [53] also showed that there were no substantial changes in methylation at the IG-DMR in the TET-KO ES cells as compared to wild-type ES cells, consistent with our observation that there is very little 5-hmC at the IG-DMR in embryos and neonatal liver (Table 3). Indeed, the IG-DMR has a very stable allele-specific DNA methylation pattern, in accordance with its role as an imprinting control element critical for normal growth and development [9, 54]. In contrast, the consistently higher levels of 5-hmC across we observed across development at the *Dlk1*-DMR suggests that 5-hmC may be a more stable epigenetic mark as this locus, as it is in the developing brain [data herein; 55].

We have yet to determine whether the high levels of hemimethylation at secondary DMRs plays a functional role. Arand et al. [56] illustrated a correlation between the reduction in DNA methylation levels and concomitant increase in hemimethylation levels during embryogenesis and primordial germ cell development, and suggested that hemimethylation plays a role in impairing maintenance methylation in order to keep methylation levels low. Similarly, Jin et al. [57] suggested that TET1 may act to reduce DNA methylation levels at hypomethylated CpG islands. Therefore, it would be interesting to look at allele-specific distribution of 5-hmC at imprinted loci to see whether TETs play a role in keeping unmethylated DMRs unmethylated. The assay we used for the 5-hmC studies described herein was not allele-specific, as that would have required a strain-specific polymorphism in close proximity to an *Msp*I site within the regions of analysis. The development of methods to assess 5-hmC in an allele-specific way would allow us to address this question in order to determine the significance of this epigenetic modification.

## Conclusions

Secondary DMRs associated with imprinted loci have low DNA methylation fidelity as compared to primary DMRs that serve as imprinting control regions. Our current analyses illustrate that the variable DNA methylation patterns at secondary DMRs correlate with high levels of hemimethylation at CpG dyads and high levels of 5-hmC. Our work therefore supports the hypothesis that secondary DMRs have a unique epigenetic profile that distinguishes them from primary DMRs. Our studies further provide insight into the molecular mechanisms responsible for methylation instability at secondary DMRs, as oxidation of 5-mC to 5-hmC by the TET enzymes ultimately leads to a loss of DNA methylation passively due to reduced DNMT1 fidelity and/or actively via further oxidation followed by DNA repair. These results are significant as they highlight the complexities associated with the maintenance of the epigenetic profile at secondary DMRs: Differential DNA methylation is maintained at these loci despite activities that function to reduce methylation levels. Further investigation is warranted to understand how parent of origin-specific DNA methylation is established and maintained at secondary DMRs.

## Methods

### Mice

C57BL/6J (B6) and *Mus musculus castaneus* (CAST) mice were purchased from the Jackson Laboratory. To facilitate the isolation of F_1_ hybrid mice, a strain of mice that served as the source of the *M. m. castaneus* allele (CAST12) was constructed as previously described [28]. Natural matings between B6 and CAST were used to generate F_1_ hybrid males for spermatozoa collection; all other F_1_ hybrid tissues used for bisulfite analyses were generated from natural matings between B6 and CAST12 mice. For all F_1_ hybrid tissues, the maternal allele is located on the left.

### DNA purification and bisulfite analysis

DNA was isolated from 7.5 d.p.c. embryos using the DNeasy Blood & Tissue Kit (Qiagen, Germantown, MD, cat#69504). DNA was isolated from 9.5 to 14.5 d.p.c. embryos and from 5 d.p.p. and adult liver following proteinase K digestion and a series of phenol/chloroform extractions as described previously [58]. Prior to bisulfite mutagenesis, complementary strands of DNA were covalently attached as follows: for IG-DMR analyses, 0.5 µg of genomic DNA was digested with 1 µl *Spe*I (NEB, Ipswich, MA, cat#R0133) and ligated to 1 µg of phosphorylated hairpin linker IG-DMR-HP (5′-CTAGAGCGATGCGTTCGAGCATCGCT-3′) [59]; for *Gtl2*-DMR analyses, 0.5 µg of genomic DNA was digested with 1 µl *Ban*I (NEB, Ipswich, MA, cat#R0118) and ligated to 1 µg of phosphorylated hairpin linker Gtl2-HP-3 (5′-GTACAGCGATGCGTTCGAGCATCGCT-3′). 0.5 µg of hairpin-linked, ligated DNA was denatured by incubating in freshly prepared 3 M NaOH for 20 min at 42 °C, and then subjected to bisulfite mutagenesis using an EZ DNA Methylation-Direct kit (Zymo Research, Irvine, CA, cat#D5020). All mutagenized DNAs were subjected to multiple independent PCR amplifications to ensure analysis of different strands of DNA; subclones derived from independent PCR amplifications are distinguished by different letters of the alphabet. PCR contamination was ruled out via analysis of no template negative control amplifications for both the first and second rounds of PCR. Data from multiple independent tissue samples derived from the same developmental stage were combined, as we did not detect variation between biological replicates. Primer pairs used for nested amplification of the mutagenized DNA were designed to incorporate at least one SNP as well as CpG dinucleotides within the previously analyzed DMRs [28]. All base pair numbers are from GenBank Accession Number NC_000078.6. Primers and PCR cycling conditions for the IG-DMR and for two adjacent regions within the *Gtl2*-DMR are detailed in Additional file 7: Table S6. Expected second round PCR products for the IG-DMR and the two regions of the *Gtl2*-DMR are 412 bp, 721 bp, and 695 bp, respectively. Subcloning of amplified products was achieved using a pGEM-T Easy vector (Promega Corporation, Madison, WI, cat#A1360). Sequencing reactions were outsourced to Genewiz (South Plainfield, NJ) or were performed using a Thermo Sequenase Cycle Sequencing Kit (Affymetrix, Cleveland, OH, cat#78500) and analyzed on a 4300 DNA Analyzer (LI-COR Biosciences, Lincoln, NE). Sequence polymorphisms used to distinguish B6 *vs.* CAST DNA (B/C): IG-DMR, G/A at bp#109,528,369; *Gtl2*-DMR, G/A at bp#109,541,531, A/G at bp#109,541,671, AA/GC at bp#109,541,820-109,541,821. Percent methylation was calculated based on data obtained from both complementary strands. Percent hemimethylation was calculated by dividing the number of hemimethylated CpG dyads by the number of hemimethylated plus homomethylated CpG dyads.

### 5-hydroxymethylation analysis

For 5-hydroxymethylation analyses, DNA was isolated from 9.5 d.p.c. embryos, 14.5 d.p.c. embryos, 5 d.p.p. liver and adult liver as described above. DNA derived from three different genetic backgrounds [C57BL/6 J, B6x(CAST or CAST12) and (CAST or CAST12)xB] was used to ensure that genetic background did not affect the outcome. In addition, DNA was isolated from wild-type and TET triple knockout embryonic stem cells [37]. 5-hydroxymethylation levels were assessed using an EpiMark 5-hmC and 5-mC Analysis Kit (NEB, Ipswich, MA, cat#E3317). Briefly, 2.5 µg genomic DNA or 2 µg ES cell DNA was glucosylated using 30 units of T4 ß-glucosyltransferase at 37 °C overnight. Glucosylated and unglucosylated control DNAs were treated with *Msp*I, *Hpa*II or no restriction endonuclease at 37 °C overnight. Following treatment with proteinase K, products were amplified via PCR and quantitative PCR (StepOnePlus, Applied Biosystems). Primers and PCR cycling conditions used are detailed in Additional file 8: Table S7. qPCR was performed in triplicate for each of three independent biological samples. Amount of 5-mC and 5-hmC in each sample was calculated according to Magalhães et al. [36]. Genomic coordinates for *Msp*I/*Hpa*II sites are: *Dlk1*-DMR-A, bp#109,459,830; *Dlk1*-DMR-B, bp#109,460,017; IG-DMR, bp#109,528,624; *Gtl2*-DMR-A, bp#109,541,314; *Gtl2*-DMR-B, bp#109,541,776; *Gtl2*-DMR-C, bp#109,541,811.

## Additional files

**Additional file 1.** Data used for statistical analyses of DNA methylation levels at the *Gtl2*- and IG-DMRs. The numerical data used to perform Mann-Whitney U tests and the resulting P values are contained in this file. Data from the *Gtl2*-DMR 5’ region, *Gtl2*-DMR 3’ region and the IG-DMR are presented in separate sheets. Within each sheet, data from each of the developmental stages are presented in chronological order, as they are in the Results, Figures, and Tables. Each sheet presents the information for a specific locus, tissue, cross (maternal allele x paternal allele), and parental allele analyzed, as indicated in columns A-D. % methylation (column F) was calculated by dividing the number of methylated CpG sites observed in a given subclone (column E) by the total number of CpG sites analyzed within the subclone; the raw data used to make these calculations are found in Figs. 2, 3 and 4. For the *Gtl2*-DMR 3’ region and the IG-DMR, P values were calculated independently for BxC samples vs. CxB samples. In addition, P values were calculated for the combined BxC + CxB samples, as some of the BxC and CxB sample sizes were too small to accurately perform Mann-Whitney U tests. Data presented in sheets labelled “grouped” combine subclones derived from the same PCR with identical DNA methylation patterns and identical sequences as a single sample, while every subclone analyzed is treated as an independent sample in sheets labelled “ungrouped”.

**Additional file 2: Table S1.** Percent methylation on paternal and maternal alleles at DMRs across four developmental stages.

**Additional file 3: Table S2.** Comparison of percent methylation at the Gtl2-DMR with like subclones grouped vs. ungrouped.

**Additional file 4: Table S3.** Comparison of percent methylation at the IG-DMR with like subclones grouped vs. ungrouped.

**Additional file 5: Table S4.** Percent 5-hydroxymethylcytosine at the Dlk1-, IG- and Gtl2-DMRs at four developmental stages.

**Additional file 6: : Table S5.** Quantification of bisulfite conversion failure.

**Additional file 7: Table S6.** Primer and PCR cycling conditions for amplification of bisulfite-mutagenized DNA.

**Additional file 8: Table S7.** Primers and PCR cycling conditions for 5-hmC analyses.

## Abbreviations

ICR: imprinting control region
DMR: differentially methylated region
IG-DMR: intergenic DMR
d.p.c.: days post-coitum
d.p.p.: days postpartum
B6: C57BL/6
C or CAST: *Mus musculus castaneus*
C12 or CAST12: *Mus musculus castaneus* chromosome 12 on a C57BL/6 background
PCR: polymerase chain reaction
5-mC: 5-methylcytosine
5-hmC: 5-hydroxymethylcytosine
KO: knockout
