# Supplementary material for: Asymmetric DNA methylation of CpG dyads is a feature of secondary DMRs associated with the Dlk1/Gtl2 imprinting cluster in mouse
Source: Epigenetics Chromatin. 2017 Jun 21;10:31. doi: 10.1186/s13072-017-0138-0 (PMC5480104; doi:10.1186/s13072-017-0138-0)
Supplement: Supplementary file 2 — Additional file 2: Table S1. Percent methylation on paternal and maternal alleles at DMRs across four developmental stages. [file 13072_2017_138_MOESM2_ESM.docx]

**Table S1.** Percent methylation on paternal and maternal alleles at DMRs across four developmental stages.

|  |  |  | *Dlk1*-DMR | | IG-DMR | | *Gtl2*-DMR, 5’ | *Gtl2*-DMR, 3’ | |
| --- | --- | --- | --- | --- | --- | --- | --- | --- | --- |
|  |  |  | BxC | CxB | BxC | CxB | BxC | BxC | CxB |
| 7.5 d.p.c. embryo | % methylation | P | 34.9%  (67/192) | ND | 94.2%  (912/968) | 93.5%  (616/659) | 82.2%  (217/264) | 81.8%  (18/22) | 83.6%  (92/110) |
|  |  | M | 9.7%  (31/320) | ND | 1.5%  (2/132) | 7.2%  (19/264) | 11.7%  (25/214) | 2.1%  (7/330) | 16.5%  (29/176) |
|  | % homomethylation | P | 97.1%  (33/34) | ND | 94%  (442/470) | 93.4%  (297/318) | 83.9%  (99/118) | 100%  (9/9) | 84%  (42/50) |
|  |  | M | 72.2%  (13/18) | ND | 0%  (0/2) | 58.3%  (7/12) | 0%  (0/25) | 16.6%  (1/6) | 61.1%  (11/18) |
|  | % hemimethylation | P | 2.9%  (1/34) | ND | 6%  (28/470) | 6.6%  (21/318) | 16.1%  (19/118) | 0%  (0/9) | 16%  (8/50) |
|  |  | M | 27.8%  (5/18) | ND | 100%  (2/2) | 41.7%  (5/12) | 100%  (25/25) | 83.3%  (5/6) | 38.9%  (7/18) |
| 14.5 d.p.c. embryo | % methylation | P | 27.6%  (123/446) | 21.2%  (124/585) | 96.9%  (597/616) | 97.3%  (728/748) | 82.5%  (235/285) | 97.7%  (43/44) | 84.5%  (223/264) |
|  |  | M | 4.7%  (25/536) | 3%  (8/271) | 11.1%  (39/352) | 3.8%  (5/132) | 1.5%  (2/132) | 6.1%  (27/440) | 6.6%  (13/198) |
|  | % homomethylation | P | 62.7%  (47/75) | 54.4%  (43/79) | 93.8%  (289/308) | 95.2%  (355/373) | 78.6%  (103/131) | 95.5%  (21/22) | 85.8%  (103/120) |
|  |  | M | 19%  (4/21) | 16.7%  (1/6) | 5.4%  (2/37) | 0%  (0/5) | 0%  (0/2) | 28.6%  (6/21) | 8.3%  (1/12) |
|  | % hemimethylation | P | 37.3%  (28/75) | 45.6%  (36/79) | 6.2%  (19/308) | 4.8%  (18/373) | 21.4%  (28/131) | 4.5%  (1/22) | 14.2%  (17/120) |
|  |  | M | 81%  (17/21) | 83.3%  (5/6) | 94.6%  (35/37) | 100%  (5/5) | 100%  (2/2) | 71.4%  (15/21) | 91.7%  (11/12) |
| 5 d.p.p. liver | % methylation | P | 45.3%  (115/254) | 61.6%  (138/224) | 96.8%  (852/880) | 94.3%  (1120/1188) | 69.7%  (92/132) | 90.9%  (160/176) | 84.4%  (483/572) |
|  |  | M | 10.7%  (40/373) | 23.7%  (84/354) | 17.4%  (45/258) | 1.1%  (1/88) | 8%  (14/176) | 12%  (29/242) | 15.9%  (7/44) |
|  | % homomethylation | P | 82.3%  (51/62) | 82.9%  (63/76) | 93.6%  (412/440) | 93.6%  (528/592) | 61.4%  (35/57) | 83.9%  (73/87) | 80.9%  (216/267) |
|  |  | M | 73.9%  (17/23) | 46%  (23/50) | 76%  (19/25) | 0%  (0/1) | 27.3%  (3/11) | 31.8%  (7/22) | 0%  (0/7) |
|  | % hemimethylation | P | 17.7%  (11/62) | 17.1%  (13/76) | 6.4%  (28/440) | 10.8%  (64/592) | 38.6%  (22/57) | 16.1%  (14/87) | 19.1%  (51/267) |
|  |  | M | 26.1%  (6/23) | 54%  (27/50) | 24%  (6/25) | 100%  (1/1) | 72.7%  (8/11) | 68.2%  (15/22) | 100%  (7/7) |
| adult liver | % methylation | P | 66.8%  (205/307) | 58.4%  (279/478) | 97.2%  (599/616) | 98.1%  (518/528) | 78.3%  (155/198) | 96.6%  (85/88) | 90.1%  (80/88) |
|  |  | M | 54%  (155/287) | 41.2%  (275/667) | 13.6%  (24/176) | 37.9%  (50/132) | 15.4%  (23/149) | 5.9%  (13/219) | 2.0%  (10/505) |
|  | % homomethylation | P | 84.1%  (90/107) | 72.7%  (117/161) | 95.8%  (293/306) | 97.7%  (256/262) | 72.2%  (65/90) | 93.2%  (41/44) | 81.8%  (36/44) |
|  |  | M | 69.2%  (63/91) | 73.9%  (116/157) | 71.4%  (10/14) | 92.3%  (24/26) | 21.1%  (4/19) | 85.7%  (6/7) | 25%  (2/8) |
|  | % hemimethylation | P | 15.9%  (17/107) | 27.3%  (44/161) | 4.2%  (13/306) | 2.3%  (6/262) | 27.8%  (25/90) | 6.8%  (3/44) | 18.2%  (8/44) |
|  |  | M | 30.8%  (28/91) | 26.1%  (41/157) | 28.6%  (4/14) | 7.7%  (2/26) | 78.9%  (15/19) | 14.3%  (1/7) | 75%  (6/8) |
|  | % methylation | P | 42.5%  (510/1199) | 42%  (541/1287) | 96.1%  (2960/3080) | 95.5%  (2982/3123) | 79.5%  (699/879) | 92.7%  (306/330) | 85.9%  (878/1034) |
|  |  | M | 16.6%  (251/1516) | 28.4%  (367/1292) | 11.2%  (110/918) | 12.2%  (75/616) | 9.5%  (64/671) | 6.2%  (76/1231) | 6.4%  (59/923) |
|  |  | total | 28%  (761/2715) | 35.2%  (908/2579) | 76.8%  (3070/3998) | 81.8%  (3057/3739) | 49.2%  (763/1550) | 24.5%  (382/1561) | 47.9%  (937/1957) |
|  | % homomethylation | P | 79.5%  (221/278) | 70.6%  (223/316) | 94.2%  (1436/1524) | 92.9%  (1436/1545) | 76.3%  (302/396) | 88.9%  (144/162) | 82.5%  (397/481) |
|  |  | M | 63.4%  (97/153) | 65.7%  (140/213) | 39.7%  (31/78) | 70.5%  (31/44) | 12.2%  (7/57) | 35.7%  (20/56) | 31.1%  (14/45) |
|  |  | total | 73.8%  (318/431) | 68.6%  (363/529) | 91.6%  (1467/1602) | 92.3%  (1467/1589) | 68.2%  (309/453) | 75.2%  (164/218) | 78.1%  (411/526) |
|  | % hemimethylation | P | 20.5%  (57/278) | 29.4%  (93/316) | 5.8%  (88/1524) | 7.1%  (109/1545) | 23.7%  (94/396) | 11.1%  (18/162) | 17.5%  (84/481) |
|  |  | M | 36.6%  (56/153) | 34.3%  (73/213) | 60.3%  (47/78) | 29.5%  (13/44) | 87.7%  (50/57) | 64.3%  (36/56) | 68.9%  (31/45) |
|  |  | total | 26.2%  (113/431) | 31.4%  (166/529) | 8.4%  (135/1602) | 7.7%  (122/1589) | 31.8%  (144/453) | 24.8%  (54/218) | 21.9%  (115/526) |

Percent methylation at CpG dinucleotides (# methylated cytosines/total # CpG dinucleotides) and percent homomethylation and hemimethylation at CpG dyads (# homomethylated CpG dyads/# homomethylated + hemimethylated CpG dyads or # hemimethylated CpG dyads/# homomethylated + hemimethylated CpG dyads). Data are reported for paternal (P) *vs.* maternal (M) alleles from BxCAST12 (BxC) *vs*. CAST12xB (CxB) F_1_ hybrids at four developmental stages. Averages across all four developmental stages are presented at the bottom of the table. Data for the *Dlk1*-DMR were calculated from Gagne *et al*. (2014). ND = not determined.
