# Supplementary material for: Asymmetric DNA methylation of CpG dyads is a feature of secondary DMRs associated with the Dlk1/Gtl2 imprinting cluster in mouse
Source: Epigenetics Chromatin. 2017 Jun 21;10:31. doi: 10.1186/s13072-017-0138-0 (PMC5480104; doi:10.1186/s13072-017-0138-0)
Supplement: Supplementary file 3 — Additional file 3: Table S2. Comparison of percent methylation at the Gtl2-DMR with like subclones grouped vs. ungrouped. [file 13072_2017_138_MOESM3_ESM.docx]

**Table S2.** Comparison of percent methylation at the *Gtl2*-DMR with like subclones grouped vs. ungrouped.

|  |  |  | *Gtl2*-DMR, 5’, BxC  grouped | | P value | Gtl2-DMR, 5’, BxC  ungrouped | | P value |
| --- | --- | --- | --- | --- | --- | --- | --- | --- |
| 7.5 d.p.c. embryo | % methylation | P | 82.2% | (217/264) | 0.0001 | 83.4% | (257/308) | <0.0001 |
|  |  | M | 11.7% | (25/214) |  | 9.2% | (32/346) |  |
|  | % homomethylation | P | 83.9% | (99/118) |  | 86.2% | (119/138) |  |
|  |  | M | 0% | (0/25) |  | 0% | (0/30) |  |
|  | % hemimethylation | P | 16.1% | (19/118) |  | 13.8% | (19/138) |  |
|  |  | M | 100% | (25/25) |  | 100% | (30/30) |  |
| 14.5 d.p.c. embryo | % methylation | P | 82.5% | (235/285) | 0.0024 | 84.8% | (279/329) | 0.0001 |
|  |  | M | 1.5% | (2/132) |  | 0.9% | (2/220) |  |
|  | % homomethylation | P | 76.8% | (103/131) |  | 81.7% | (125/153) |  |
|  |  | M | 0% | (0/2) |  | 0% | (0/2) |  |
|  | % hemimethylation | P | 21.4% | (28/131) |  | 18.3% | (28/153) |  |
|  |  | M | 100% | (2/2) |  | 100% | (2/2) |  |
| 5 d.p.p. liver | % methylation | P | 69.7% | (92/132) | 0.0024 | 69.7% | (92/132) | 0.0014 |
|  |  | M | 8% | (14/176) |  | 6.4% | (14/220) |  |
|  | % homomethylation | P | 61.4% | (35/37) |  | 61.4% | (35/37) |  |
|  |  | M | 27.3% | (3/11) |  | 27.3% | (3/11) |  |
|  | % hemimethylation | P | 38.6% | (22/57) |  | 38.6% | (22/57) |  |
|  |  | M | 72.7% | (8/11) |  | 72.7% | (8/11) |  |
| adult liver | % methylation | P | 78.3% | (155/198) | 0.0021 | 78.3% | (155/198) | 0.0004 |
|  |  | M | 15.4% | (23/149) |  | 9.7% | (23/237) |  |
|  | % homomethylation | P | 72.2% | (65/90) |  | 72.2% | (65/90) |  |
|  |  | M | 21.1% | (4/19) |  | 21.1% | (4/19) |  |
|  | % hemimethylation | P | 27.8% | (25/90) |  | 27.8% | (25/90) |  |
|  |  | M | 78.9% | (15/19) |  | 78.9% | (15/19) |  |
|  | % methylation | P | 79.5% | (699/879) |  | 81% | (783/967) |  |
|  |  | M | 9.5% | (64/671) |  | 6.9% | (71/1023) |  |
|  |  | total | 49.2% | (763/1550) |  | 42.9% | (854/1990) |  |
|  | % homomethylation | P | 76.3% | (302/396) |  | 78.5% | (344/438) |  |
|  |  | M | 12.2% | (7/57) |  | 11.3% | (7/62) |  |
|  |  | total | 68.2% | (309/453) |  | 70.2% | (351/500) |  |
|  | % hemimethylation | P | 23.7% | (94/396) |  | 21.5% | (94/438) |  |
|  |  | M | 87.7% | (50/57) |  | 88.7% | (55/62) |  |
|  |  | total | 31.8% | (144/453) |  | 29.8% | (149/500) |  |

Percent methylation at CpG dinucleotides (# methylated cytosines/total # CpG dinucleotides) and percent homomethylation and hemimethylation at CpG dyads (# homomethylated CpG dyads/# homomethylated + hemimethylated CpG dyads or # hemimethylated CpG dyads/# homomethylated + hemimethylated CpG dyads). Data are reported for paternal (P) *vs.* maternal (M) alleles from BxCAST12 (BxC) F_1_ hybrids at four developmental stages. Averages across all four developmental stages are presented at the bottom of the table. “Grouped” column reports data when subclones from the same PCR with identical methylation patterns are grouped; “ungrouped” column reports data when subclones from the same PCR with identical methylation patterns are analyzed as independent samples.
