# Supplementary material for: Asymmetric DNA methylation of CpG dyads is a feature of secondary DMRs associated with the Dlk1/Gtl2 imprinting cluster in mouse
Source: Epigenetics Chromatin. 2017 Jun 21;10:31. doi: 10.1186/s13072-017-0138-0 (PMC5480104; doi:10.1186/s13072-017-0138-0)
Supplement: Supplementary file 5 — Additional file 5: Table S4. Percent 5-hydroxymethylcytosine at the Dlk1-, IG- and Gtl2-DMRs at four developmental stages. [file 13072_2017_138_MOESM5_ESM.docx]

**Table S4.** Percent 5-hydroxymethylcytosine at the *Dlk1*-, IG- and *Gtl2*-DMRs at four developmental stages.

|  | *Dlk1*-DMR, site A | *Dlk1*-DMR, site B | IG-DMR | *Gtl2*-DMR, site A | *Gtl2*-DMR, site B | *Gtl2*-DMR, site C |
| --- | --- | --- | --- | --- | --- | --- |
| 9.5 d.p.c embryo | B, 21%  B, 21%  BxC, 19%  CxB, 25% | B, 7.5%  BxC, 3.7%  CxB, 3.5% | B, 3.8%  BxC, 3.2%  CxB, 1.6% | B, 0%  B, 0%  BxC, 0%  CxB, 0% | B, 1.3%  B, 2%  BxC, 0.9%  CxB, 1.4% | B, 0.79% |
| 9.5 d.p.c. average (n) | 21.5% (4) | 6.6% (3) | 2.9% (3) | 0% (4) | 1.4% (4) |  |
| 14.5 d.p.c. embryo | B, 40%  BxC, 39.5%  CxB, 37.6% | BL/6, 10%  BxC, 10.7%  CxB, 9.3% | B, 5%  BxC, 2.1%  CxB, 2.3% | BL/6, 1%  BxC, 0%  CxB, 1.5% | B, 1.5%  BxC, 1.5%  CxB, 1.9% | B, 1.2% |
| 14.5 d.p.c. average (n) | 39% (3) | 10% (3) | 3.1% (3) | 0.8% (3) | 1.6% (3) |  |
| 5 d.p.p. liver | B, 8%  B, 9%  BxC, 18.5%  BxC, 9.2%  BxC, 9%  CxB, 8.5%  CxB, 9.4%  CxB, 9.9% | B, 3.4%  B, 4.7%  BxC, 6.9%  BxC, 7.3%  BxC, 11.1%  CxB, 4.4%  CxB, 4.2%  CxB, 9.9% | B, 4.4%  BxC, 3.3%  CxB, 3.5% | B, 0.02%  B, 5.4%  BxC, 3.1%  CxB, 1.4% | B, 0.07%  B, 0%  BxC, 1.5%  BxC, 1.2%  CxB, 1%  CxB, 1.7% | B, 1.4% |
| 5 d.p.p. liver average (n) | 10.2% (8) | 6.5% (8) | 3.7% (3) | 2.5% (4) | 0.9% (6) |  |
| adult liver | B, 5.7%  BxC, 7.6%  CxB, 10.9% | BL/6, 2%  BxC, 2.4%  CxB, 0.7% | B, 5.3%  BxC, 8.1%  CxB, 12.8% | B, 0.1%  BxC, 1.8%  CxB, 3.8% | B, 0%  BxC, 1.1%  CxB, 0% | B, 2.4% |
| adult liver average (n) | 8.1% (3) | 1.7% (3) | 8.7% (3) | 1.9% (3) | 0.4% (3) |  |

Data are reported for three different genetic backgrounds: C57BL/6J (B) and BxCAST12 (BxC) and CAST12xB (CxB) F_1_ hybrids. For *Gtl2*-DMR site C, only C57BL/6J data are reported as a strain-specific polymorphism abolishes this *Msp*I site in *Mus musculus castaneus.* Average 5-hydroxymethylcytosine across all three genetic backgrounds are also reported. Amount of 5-mC and 5-hmC in each sample was calculated according to Magalhaes *et al*. (2013).
