# Supplementary material for: Asymmetric DNA methylation of CpG dyads is a feature of secondary DMRs associated with the Dlk1/Gtl2 imprinting cluster in mouse
Source: Epigenetics Chromatin. 2017 Jun 21;10:31. doi: 10.1186/s13072-017-0138-0 (PMC5480104; doi:10.1186/s13072-017-0138-0)
Supplement: Supplementary file 6 — Additional file 6: : Table S5. Quantification of bisulfite conversion failure. [file 13072_2017_138_MOESM6_ESM.docx]

**Table S5.** Quantification of bisulfite conversion failure.

| **region analyzed** | | **# non-CpG cytosines** | **failed conversion (n)** | | **failed conversion (%)** | |
| --- | --- | --- | --- | --- | --- | --- |
| *Gtl2*-DMR, 5’ | 7,526 | | | 66 | | 0.88% |
| *Gtl2*-DMR, 3’ | 4,998 | | | 49 | | 0.98% |
| IG-DMR | 8,344 | | | 176 | | 2.11% |
| total | 20,868 | | | 291 | | 1.39% |

Failed conversion was assessed by counting the number of cytosines present at non-CpG sites in bisulfite-treated DNA; the total number of failed conversion events was divided by the total number of non-CpG cytosines analyzed to calculate the failed conversion rate.
