# Supplementary material for: Asymmetric DNA methylation of CpG dyads is a feature of secondary DMRs associated with the Dlk1/Gtl2 imprinting cluster in mouse
Source: Epigenetics Chromatin. 2017 Jun 21;10:31. doi: 10.1186/s13072-017-0138-0 (PMC5480104; doi:10.1186/s13072-017-0138-0)
Supplement: Supplementary file 7 — Additional file 7: Table S6. Primer and PCR cycling conditions for amplification of bisulfite-mutagenized DNA. [file 13072_2017_138_MOESM7_ESM.docx]

**Table S6.** Primer and PCR cycling conditions for amplification of bisulfite-mutagenized DNA.

| DMR analyzed | round | primers | cycling conditions |
| --- | --- | --- | --- |
| IG-DMR  genomic coordinates for first and last CpG analyzed:  bp#109,528,345-  109,528,471  (NC_000078.6) | 1^st^ | 5’-TTTAATTTGTGAGAATGTTTTAGTTA-3’  5’-AAACTACAATCTATATAATCACAAC-3’ | 94°C 2 min  55°C 1 min  72°C 1 min  repeat 2x  94°C 30 sec  55°C 1 min  72°C 1 min  repeat 30x  72°C 10 min |
|  | 2^nd^ | 5’-ATTTTAGGAGTTAAGGAAAAGAAAG-3’  5’-GCTCCATTAACAAAATAATACAACCCTTCC-3’ | 94°C 30 sec  55°C 1 min  72°C 1 min  repeat 35x  72°C 10 min |
| *Gtl2*-DMR  CpG 5-15  genomic coordinates for first and last CpG analyzed:  bp#109,541,256-  109,541,514  (NC_000078.6) | 1^st^ | 5’-GTTTGAAAGGATGTGTAAAAATG-3’  5’-CCAATACTAAAAACCTTCTCCCA-3’ | 94°C 2 min  60°C 1 min  72°C 1 min  repeat 2x  94°C 30 sec  60°C 1 min  72°C 1 min  repeat 30x  72°C 10 min |
|  | 2^nd^ | 5’-GAAAGGTTAGTGTTGGGGATTT-3’  5’-CCTTAACCTAATCCCCAACATC-3’ | 94°C 30 sec  60°C 1 min  72°C 1 min  repeat 30x  72°C 10 min |
| *Gtl2*-DMR  CpG 16-29  genomic coordinates for first and last CpG analyzed:  bp#109,541,560-  109,541,812  (NC_000078.6) | 1^st^ | 5’-GGTAAAAGGTGATTTATTAAAAGTT-3’  5’-CAACACTCAAATCACCCCCC-3’ | 94°C 2 min  55°C 1 min  72°C 1.5 min  repeat 2x  94°C 30 sec  55°C 1 min  72°C 1.5 min  repeat 30x  72°C 10 min |
|  | 2^nd^ | 5’-AGTATTTAGATTATTTTTTATATTTTTATA-3’  5’-GCCCCCCACATCTATTCTACC-3’ | 94°C 30 sec  52°C 1 min  72°C 1.5 min  repeat 30x  72°C 10 min |
