# Supplementary material for: Asymmetric DNA methylation of CpG dyads is a feature of secondary DMRs associated with the Dlk1/Gtl2 imprinting cluster in mouse
Source: Epigenetics Chromatin. 2017 Jun 21;10:31. doi: 10.1186/s13072-017-0138-0 (PMC5480104; doi:10.1186/s13072-017-0138-0)
Supplement: Supplementary file 8 — Additional file 8: Table S7. Primers and PCR cycling conditions for 5-hmC analyses. [file 13072_2017_138_MOESM8_ESM.docx]

**Table S7.** Primers and PCR cycling conditions for 5-hmC analyses.

| region | primers | PCR cycling conditions | PCR product  size |
| --- | --- | --- | --- |
| *Dlk1*-DMR-A | F: 5’- GATTCGTCGACAAGACCTG-3’  R: 5’- CTTGCACAGACACTCGAA-3’ | 94°C, 30 sec  60°C, 1 min  72°C, 1 min  repeat 30x  72°C, 10 min | 113 bp |
| *Dlk1*-DMR-B | F: 5’- GGCTATGGGCTCACCTA-3’  R: 5’- GCTCTTTCATGGACACCTT-3’ |  | 97 bp |
| IG-DMR | F: 5’- ATTCTCACAGATTGGGAATGG-3’  R: 5’- CATCAGGAATTCCAAAGCTAAAC-3’ |  | 97 bp |
| *Gtl2*-DMR-A | F: 5’- CCAGCATCCAACACGAAA-3’  R: 5’- TCTTAATCAGGGTCTCTACCG-3’ |  | 133 bp |
| *Gtl2*-DMR-B | F: 5’- GTAACCATTACTAGCCGTTTCCT-3’  R: 5’- GCAGCCTTCTCTGTGATCTG-3’ |  | 104 bp |
| *Gtl2*-DMR-C | F: 5’- CCCAGATCACAGAGAAGGC-3’  R: 5’- TTCCTTCCTATGAGACGCAAG -3’ |  | 107 bp |
